# Isolation and Structural Characterisation of Okara Polysaccharides

**DOI:** 10.3390/molecules17010753

**Published:** 2012-01-13

**Authors:** Bo Li, Fei Lu, Haijuan Nan, Yang Liu

**Affiliations:** School of Food Science, Henan Institute of Science and Technology, Xinxiang 453003, Henan, China; Email: lufei709@yahoo.com.cn (F.L.); nanhaijuan1@163.com (H.N.); liuyang3212605@163.com (Y.L.)

**Keywords:** okara, polysaccharide, structure, isolation

## Abstract

Okara is a byproduct generated during tofu or soymilk production processes. Crude polysaccharide (yield 56.8%) was isolated by removing fat, protein and low molecular weight carbohydrates from initial okara. Crude okara polysaccharide was further divided into four soluble fractions and an insoluble residue fraction by extracting with 0.05 M EDTA + NH_4_ oxalate, 0.05 M NaOH, 1 M NaOH and 4 M NaOH, with yields of 7.7%, 3.6%, 20.7%, 16.0% and 27.9%, respectively. Arabinose, galactose, galacturonic acid, xylose and glucose (only for the insoluble fraction) were the major constituent sugars. The primary sugar residues of okara polysaccharides were 1,4-linked β-galactopyranose, 1,5- and 1,3-linked α-arabinofuranose, 1,5-linked α-xylofuranose, 1,2-linked, 1,2,4-linked and terminal α-rhamnopyranose (or fucopyranose), and 1,4-linked β-glucopyranose (only for the insoluble fraction), indicating okara polysaccharides might contain galactan, arabinan, arabinogalactan, xylogalacturonan, rhamnogalacturonan, xylan, xyloglucan and cellulose.

## 1. Introduction

Okara is the residue obtained from ground soybean after removing the water-extractable fraction used to produce tofu or soymilk. About 1.2 kg of fresh okara is produced from 1 kg of soybean processed for manufacturing tofu [1]. Huge quantities of okara are produced worldwide. In Japan about 800,000 tons, in Korea approximately 310,000 tons, and in China about 2,800,000 tons of okara are produced from the tofu industry every year [2,3]. These huge quantities of okara produced annually pose a significant disposal problem. Okara is sometimes used as animal feed but most is dumped and burned as waste.

Polysaccharides from dietary fibre are important among functional compounds due to the well-known role that dietary fibre plays in many physiological processes and in the prevention of different diseases [4]. In recent years, there has been a trend to find new sources of dietary fibre that can be used as ingredients in the food industry. Dry okara contains about 50% dietary fibre and 25% protein, so it is an excellent dietary fibre source and as such could be added to different foods [5,6,7]. Many studies have been undertaken on chemical components, nutrition and utilization of okara [8]. However, few reports have been found in the literature on the structural characterisation of polysaccharides in okara [9,10,11]. Knowledge of the structure may promote to explain the relationship of function and structure of okara polysaccharides and allow the use of okara in different functional foods. The aim of this work was to investigate the structural characteristics of polysaccharides from okara through sequential extraction with different extractive solutions.

## 2. Results and Discussion

### 2.1. Isolation and Fractionation of Okara Polysaccharides

Generally, okara contains about 50% dietary fibre, 25% protein, 10% lipid, 4% low molecular weight carbohydrates and 4% ash [1,5,6,7]. Other soy components that are also likely present in okara include isoflavones, lignans, phytosterols, coumestans, saponins and phytates. The chemical composition of okara will depend on the amount of water phase extracted from the ground soybean and whether further water was added to extract residual extractable components. It also depends on the cultivar of soybean and the production methods [8]. The okara in our study had 46.3% dietary fibre, 17.8% protein, 5.9% lipid, 3.9% ash, 2.6% reducing sugar, 0.22% flavone and 6.7% moisture.

After removing the fat, protein and low molecular weight carbohydrates from okara, the crude okara polysaccharide (COP) was obtained which accounted for 56.8% of the initial okara. COP was sequentially treated with 0.05 M ethylenediaminetetraacetic acid disodium (EDTA) + NH_4_ oxalate, 0.05 M NaOH, 1 M NaOH and 4 M NaOH to provide four soluble fractions (OP1~OP4) and an insoluble residue (OP5). The yields of OP1~OP5 were 7.7%, 3.6%, 20.7%, 16.0% and 27.9% of COP, respectively. The isolation and fractionation of okara polysaccharides have been described by Yamaguchi *et al.*, Huisman *et al.*, and Mateos-Aparicio *et al.* [9,10,11,12]. The latter obtained 77.2 g cell wall material (CWM) from 100 g okara by delivering fat, protein and low molecular weight carbohydrates. They sequentially extracted CWM with 0.05 M cyclohexanediamine tetraacetic acid (CDTA) + NH_4_ oxalate, 0.05 M NaOH, 1 M KOH, 4 M KOH and 0.3% NaClO_2_/acetic acid to leave a cellulose-rich residue, and the yields of the six fractions were 16.7%, 2.9%, 20.0%, 13.6%, 19.7% and 21.9% of CWM, respectively [11]. Their results showed that chelating agent (CDTA) and strong alkali extracted the most okara polysaccharide. The study of Huisman *et al.* revealed that chelating agent soluble solids was the major extract, yielding 38% of the polysaccharides present in the water unextractable solids from soybean meal [10]. Our study showed strong alkali soluble solids was the major extract, and the yield of chelating agent (EDTA) soluble solids just was 7.7% of okara. The difference was partly due to the okara material used. In our study, the okara was obtained by the Chinese method, and in the study of Mateos-Aparicio *et al.*, okara was obtained by the Japanese method, *i.e.*, the rehydrated soybean was cooked before grinding and filtering. In the study of Huisman *et al.*, soybean meal, the residue of oil extraction, was used as the raw material. Another reason might be the difference of chelating agent, CDTA and EDTA. Therefore, the isolation and fractionation of polysaccharides were influenced by the okara materials and extraction reagents. 

### 2.2. Monosaccharide Compositions of Okara Polysaccharides

The monosaccharide compositions of okara, crude okara polysaccharide and five polysaccharide fractions are shown in Table 1. The main neutral sugars of okara polysaccharides are arabinose, galactose and xylose, and the minor sugars are rhamnose, glucose, fucose and mannose. Okara polysaccharides also contain a considerable amount of galacturonic acid. Mateos-Aparicio *et al.* and Tsubaki *et al.* also reported galactose, arabinose and galacturonic acid as the major sugars [11,13]. However, in the study of Mateos-Aparicio *et al.*, a maximum amount of arabinose was present in the cellulose-rich residue (equivalent to OP5), and more xylose existed in the 4 M NaOH fraction [11]. 

The high values of arabinose (22~28%), galactose (14~41%), galacturonic acid (10~35%) and xylose (5~17%) are probably due to pectins and hemicelluloses. Some soybean cell wall polysaccharides have already been partly elucidated during the past years, and include arabinans, galactans, arabinogalactans, xylogalacturonans, and rhamnogalacturonans, *etc.* [10]. Because okara mainly comes from the water unextractable cell wall material of soybean, it might contain similar polysaccharides as soybean to some extent. Yamaguchi *et al.* has reported a pectic polysaccharide from okara, which comprised regions of galacturonan and rhamnogalacturonan carrying side chains composed mainly of homogeneous arabinan and galactan. The galacturonan regions were distributed at both the reducing and nonreducing ends of the polysaccharide [9].

**Table 1:** Monosaccharide compositions of okara polysaccharides.

Sample	Monosaccharide compositions (mol%)							
	Ara	Rha	Fuc	Xyl	Man	Gal	Glc	GalA
Okara	23.6	5.1	3.3	12.8	3.1	26.1	5.8	20.3
COP	27.4	4.1	2.8	13.9	2.2	29.0	2.4	18.1
OP1	24.2	3.9	1.9	5.7	2.8	24.4	1.6	35.4
OP2	28.7	6.4	3.6	10.2	nd	14.0	1.4	35.7
OP3	26.7	4.5	3.3	11.8	1.0	31.2	3.1	18.4
OP4	22.8	2.7	2.1	12.7	4.4	41.8	3.4	10.1
OP5	15.5	5.4	1.3	17.1	1.0	14.6	26.8	18.2

COP: crude okara polysaccharide; OP1: fraction extracted with EDTA-ammonium oxalate; OP2: fraction extracted with 0.05 M NaOH; OP3: fraction extracted with 1 M NaOH; OP4: fraction extracted with 4 M NaOH; OP5: residue left after sequential extraction; nd: not detected.

In terms of five polysaccharide fractions, their monosaccharide compositions have some differences. Fractions OP1 and OP2 contain more galacturonic acid (35%) in comparison with other fractions, indicating the two fractions include a large number of pectic polymers that was easily extracted by EDTA/ammonium oxalate through the formation of a chelated complex and by dilute alkali. Fractions OP3 and OP4 contain more galactose than other fractions, indicating galactans and arabinogalactans were easily extracted by strong alkali. The amounts of glucose and xylose in fraction OP5 were the highest in five the fractions, suggesting the insoluble fibre of okara might be composed of celluloses, xylans and xyloglucans. Arabinose is one of the main sugars and the amount is similar in fractions OP1~OP4, thus arabinose-containing polysaccharides are in a great quantity and almost similar in the four soluble fractions. Xylose content gradually increases from OP1 to OP5. This might be due to the xylogalacturonan regions described previously in the hull of the pea by Le Goff *et al.* [14], in apple by Schols *et al.* [15], and in soybean meal by Huisman *et al.* [10]. 

### 2.3. Methylation Analysis of Okara Polysaccharides

Methylation results (Table 2) showed that the main structural features for all okara polysaccharide fractions are →4)Hex*p*(1→ and →5)Pen*f* (1→, suggesting galactose and glucose (for OP5) residues are mainly 1,4-linked pyranoses, and arabinose and xylose residues are primary 1,5-linked furanoses based on their sugar compositions. 

**Table 2:** Methylation results of okara polysaccharide fractions.

Structural feature	COP	OP1	OP2	OP3	OP4	OP5
Pen*f*(1→	2.46	7.57	3.71	9.41	1.46	0.39
Pen*p*(1→	1.03	2.11	0.84	1.68	1.00	nd
dHex*p* (1→	1.43	3.96	2.21	1.84	2.45	0.21
→5)Pen*f* (1→	7.37	12.76	17.56	11.42	7.91	4.55
→5)Pen*f* (1→	5.27	2.78	3.07	5.29	6.20	13.65
Hex*p*(1→	2.39	4.22	4.56	2.77	3.97	0.40
→3)Pen*f*(1→	1.79	4.84	3.97	2.37	2.35	0.22
→2)dHex*p*(1→	1.01	1.31	1.85	nd	0.82	nd
→2,4)Hex*p*(1→	nd	nd	nd	nd	nd	0.43
→2,4)dHex*p*(1→	1.49	2.21	3.33	1.15	0.80	0.37
→4)Hex*p*(1→	40.47	30.94	48.67	53.07	51.87	4.31
→4)Hex*p*(1→	27.61	2.49	1.03	1.16	7.22	69.17
→2)Hex*p*(1→	nd	nd	nd	nd	1.07	nd
→6)Hex*p*(1→	0.64	nd	1.95	0.88	0.82	nd
→6)Hex*p*(1→	nd	nd	0.54	nd	nd	nd
→3,4)Hex*p*(1→	nd	nd	0.67	0.28	nd	0.40
→2,6)Hex*p*(1→	0.48	0.60	0.50	0.20	nd	0.41
→4,6)Hex*p*(1→	nd	nd	nd	0.10	1.13	nd
→4,6)Hex*p*(1→	2.12	0.92	nd	0.68	5.16	0.86
→4,6)Hex*p*(1→	0.59	0.72	1.18	nd	0.40	0.95

Pen: pentose; Hex: hexose; dHex: deoxy hexose;. *f*: furanose; *p*: pyranose; nd: not detected.

As far as OP1 is concerned, the linkage types for pentose reidues are mainly →5)Pen*f*(1→ (12.76%), Pen*f*(1→ (7.57%) and →3)Pen*f*(1→ (4.84%), indicating arabinose and xylose residues are mainly terminal, 1,5- and 1,3-linked. The primary linkage type of deoxyhexose is dHex*p* (1→, suggesting most of rhamnose (or fucose) residues locate at the nonreducing terminals of polysaccharides. The predominant linkage type for galactose residues is 1,4-linked (30.94%). According to the data of methylation analysis and sugar composition, OP1 may comprise an arabinan, containing 1,5- and 1,3-linked arabinofuranose residues; a galactan, containing 1,4-linked galactopyranose residues; a xylogalacturonan, containing 1,5-linked xylofuranose residues; and a rhamnogalacturonan, containing 1,2-linked with highly branching at C-4 rhamnopyranose residues. 

Compared with the methylation result of OP1, the main differences of OP2, OP3 and OP4 are mole ratios of Pen*f*(1→, →5)Pen*f* (1→ and →4)Hex*p*(1→. OP2 has more →5)Ara*f* (1→ and fewer Ara*f* (1→, indicating the branching degree of arabinan is lower than that of OP1. OP3 and OP4 have more →5)Xyl*f* (1→ and fewer galacturonic acid in comparison with OP1, suggesting the two fractions might contain xylans. The primary structural feature of OP5 is →4)Glc*p*(1→, indicating cellulose is the main component of OP5. Furthermore, OP5 could also contain a few of xylans and xyloglucans due to a considerable amount of →5)Xyl*f* (1→.

### 2.4. NMR of Okara Polysaccharides

**Figure 1:**
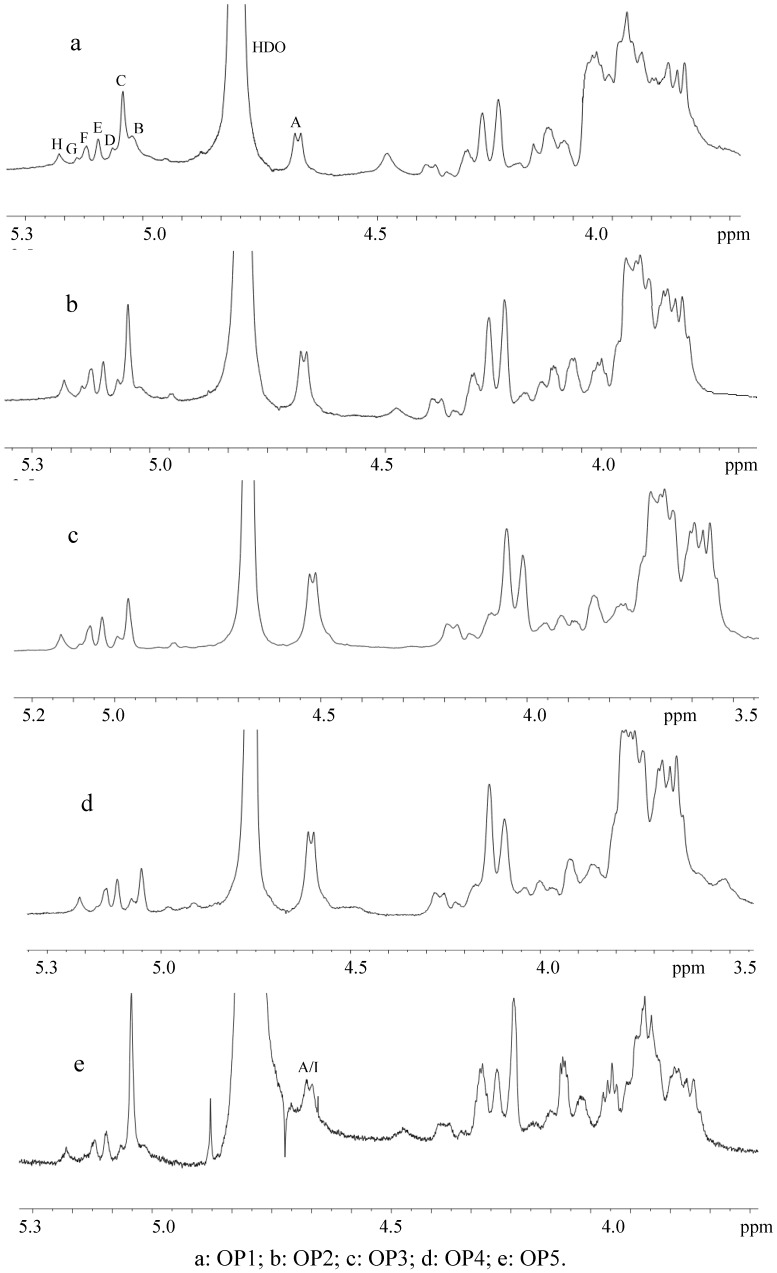
500-MHz ^1^H-NMR spectra of okara polysaccharides.

The configuration of the anomeric protons of sugar residues in polysaccharides can be identified from their ^1^H-NMR spectra according to their chemical shift (δ) and coupling constant (^3^*J*_1,2_). In generally, anomeric protons with δ > 5.0 and ^3^*J*_1,2_ < 4.0 Hz are in an α configuration, and anomeric protons with δ < 5.0 and ^3^*J*_1,2_ > 6.0 Hz are in a β configuration. The ^1^H-NMR spectra of OP1, OP2, OP3 and OP4 (Figure 1) show that the chemical shifts and ^3^*J*_1,2_ of most anomeric protons (B~H) are higher than 5.0 and lower than 4.0 Hz, suggesting these anomeric protons are of the α configuration. Anomeric proton A has a strong signal at δ 4.6, and its ^3^*J*_1,2_ is about 8.0 Hz, indicating A is in a β configuration. In terms of the sugar composition and methylation analysis of OP1~OP4, it can be deduced that A is the anomeric proton of →4)β-Gal*p*(1→ residue. Thus, it can also be inferred that arabinose, xylose, rhamnose, fucose and glucouronic acid are of the α anomeric configuration. With respect to OP5, there are at least two peaks (A, I) at δ 4.56~4.66, indicating more than two anomeric protons are β configurations. According to methylation result of OP5, it could be deduced that I is the anomeric proton of →4)β-Glc*p*(1→ residue.

Figure 1 shows the ^1^H-NMR spectra of OP1~OP4 are somewhat similar, which conforms to the sugar composition and methylation analysis data. The result suggests the polysaccharides in OP1~OP4 could be similar to some extent, and the differences might be the molecular size, branch degree, and the ratio of different sugar residues of polysaccharides. OP5 has a different ^1^H-NMR spectrum with other four fractions, which might be caused by the large amount of cellulose residues in OP5.

## 3. Experimental

### 3.1. Materials

Fresh okara was obtained from a tofu production line of Henan Xiaobao Douye Co. Ltd., Xinxiang, China. Soybean (*Glycine max* L.), north-east variety (China) was soaked, rinsed, and ground, and the okara was filtered off according to guidelines for the Chinese method. Okara was freeze-dried (LGJ-18 freeze-dryer, Beijing Sihuan) and ground to fine powder (80 mesh). Standard sugars were purchased from Sigma. The other chemicals and reagents used were of analytical grade.

### 3.2. Isolation and Sequential Extraction of Polysaccharides from Okara

Okara polysaccharide was isolated and extracted according to the methods of Huisman *et al.* and Mateos-Aparicio *et al.* [10,11]. Okara (20 g) was defatted in a Soxhlet system by extraction with diethyl ether solvent. Defatted okara was treated with 1.5% (w/v) sodium dodecyl sulfate solution containing 10 mM 1,4-dithioerythreitol for 3 h at room temperature. After centrifugation (5,000 r/min, 30 min), this extraction was repeated three times to remove the protein. The final residue was washed twice with distilled water and freeze-dried. Afterwards, the dried residue (14.40 g) were extracted with 85% ethanol for 1 h at 60 °C. After centrifugation (5,000 r/min, 30 min), this extraction was repeated twice to deliver low molecular weight carbohydrates. The residue was freeze-dried and 11.35 g crude okara polysaccharide (COP) was obtained. 

COP (10 g) was sequentially extracted with 0.05 M EDTA + 0.05 M NH_4_ oxalate in 0.05 M sodium acetate buffer, pH 5.0 (four times 300 mL) at 70 °C for 1 h to separate the OP1 fraction. The remnant was then extracted with 0.05 M NaOH, 4 °C, 1 h (OP2 fraction), 1 M NaOH + 0.02 M NaBH_4_, room temperature, 2 h (OP3 fraction) and 4 M NaOH + 0.02 M NaBH_4_, room temperature, 2 h (OP4 fraction). Each extraction was done three times. Each extract was neutralised with glacial acetic acid, concentrated at 50 °C in low-pressure, dialysed with 12–14 KDa membrane against distilled water and freeze-dried. The residue of OP4 extraction was neutralised with acetic acid, followed by centrifugation. The precipitation was taken out with distilled water and dialysed with 12–14 KDa membrane against distilled water. The remain was freeze-dried to obtain OP5 fraction. 

### 3.3. Determination of Monosaccharide Composition

Okara polysaccharide samples were subjected to methanolysis (1 M methanolic HCl, 24 h, 85 °C), after addition of internal standard (mannitol). The resulting mixtures of methyl glycosides were trimethylsilylated (hexamethyldisilazane-trimethylchlorosilane-pyridine 1:1:5, 30 min, room temperature), and then analyzed by GLC on an EC-1 column (30 m × 0.32 mm, Alltech), using a Chrompack CP9002 gas chromatograph (temperature program 140–240 °C, 4 °C/min) [16].

### 3.4. Methylation Analysis

Okara polysaccharide samples were permethylated using CH_3_I and solid NaOH in DMSO, as described earlier [17]. After hydrolysis with 2 M TFA (2 h, 120 °C), the partially methylated monosaccharides were reduced with NaBD_4_ (2 h, room temperature). Then neutralisation with HOAc and removal of boric acid by co-evaporation with methanol, followed by acetylation with 1:1 acetic anhydride: pyridine (30 min, 120 °C), a mixture of partially methylated alditol acetates were yielded, which was analysed by GLC-EI-MS on an EC-1 column (30 m × 0.25 mm) using a GCMS-QP2010. Plus instrument (Shimadzu, Japan) and temperature gradient (140–250 °C at 8 °C /min).

### 3.5. NMR Analysis

Resolution-enhanced 500-MHz ^1^H-NMR spectra of okara polysaccharide samples were recorded in D_2_O on a Varian-500 spectrometer at a probe temperature of 300 K. Prior to analysis, samples were exchanged twice in D_2_O (99.9 atom% D, Cambridge Isotope Laboratories, Inc., Andover, MA, USA) with intermediate lyophilization, and then dissolved in 0.6 mL D_2_O.

## 4. Conclusions

Okara contains 56.8% crude polysaccharides after removing fat, protein and low molecular weight carbohydrates from the initial okara. Crude okara polysaccharides can be further partitioned into five fractions by extracting with 0.05 M EDTA + NH_4_ oxalate, 0.05 M NaOH, 1 M NaOH and 4 M NaOH, and their yields were 7.7%, 3.6%, 20.7%, 16.0% and 27.9%, respectively. The main monosaccharides of okara polysaccharides are arabinose, galactose, galacturonic acid and xylose. OP5 also contains a great amount of glucose. The primary sugar residues of okara polysaccharides are 1,4-linked β-galactopyranose, 1,5- and 1,3-linked α-arabinofuranose, 1,5-linked α-xylofuranose, 1,2-linked, 1,2,4-linked and terminal α-rhamnopyranose (or fucopyranose), and 1,4-linked β-glucopyranose (only for OP5). These results indicate okara polysaccharides might contain galactan, arabinan, arabinogalactan, xylogalacturonan, rhamnogalacturonan, xylan, xyloglucan and cellulose.
